# Barometric-pumping controls fugitive gas emissions from a vadose zone natural gas release

**DOI:** 10.1038/s41598-019-50426-3

**Published:** 2019-10-01

**Authors:** Olenka N. Forde, Aaron G. Cahill, Roger D. Beckie, K. Ulrich Mayer

**Affiliations:** 1 Earth, Ocean and Atmospheric Sciences, University of British Columbia, 2020-2207 Main Mall, Vancouver, BC V6T 1Z4 Canada; 2 Heriot-Watt University, Lyell Centre, Research Avenue South, Edinburgh, EH14 4AP United Kingdom

**Keywords:** Hydrology, Environmental impact, Hydrology

## Abstract

Subsurface natural gas release from leaking oil and gas wells is a major environmental concern. Gas migration can cause aquifer contamination, explosive conditions in soil gas, and greenhouse gas emissions. Gas migration is controlled by complex interacting processes, thus constraining the distribution and magnitude of “fugitive gas” emissions remains a challenge. We simulated wellbore leakage in the vadose zone through a controlled release experiment and demonstrate that fugitive gas emissions can be directly influenced by barometric pressure changes. Decreases in barometric-pressure led to surface gas breakthroughs (>20-fold increase in <24 hours), even in the presence of low-permeability surficial soils. Current monitoring strategies do not consider the effect of barometric pressure changes on gas migration and may not provide adequate estimates of fugitive gas emissions. Frequent or continuous monitoring is needed to accurately detect and quantify fugitive gas emissions at oil and gas sites with a deep water table.

## Introduction

Global inventories suggest that at least 7% of oil and gas wells show some loss of wellbore integrity^1–3^. Wellbore failure can allow natural gas to escape into the subsurface^4–7^. Methane ([CH_4_], the primary component of natural gas) can subsequently spread laterally and vertically^8,9^ resulting in aquifer contamination^10,11^, explosive conditions in soil gas^12,13^, and emissions to the atmosphere^14–16^. To mitigate potential risks, active, abandoned and orphaned wells with gas migration need to be identified and monitored^17,18^. However, fugitive-gas is difficult to detect due to the unpredictable nature of interacting transport and reaction processes in the subsurface^19–21^. Existing techniques involve vehicle^22,23^, aircraft^24,25^ and equipment/facility^26,27^ based measurements that constrain regional-scale CH_4_ emissions in areas of oil and gas development. These studies do not identify individual wells with gas migration or the spatiotemporal distribution of emissions at a site. Currently, gas migration is not rigorously monitored at the well-pad scale. If regulators request an inspection, they rely on infrequent or one-time point-source measurements of shallow soil-gas concentrations^28–32^. These measurement methods are highly restrictive in their ability to detect and quantify emissions through time and space and almost certainly underrepresent the true magnitude of gas release at many leaking well sites^33^.

At landfills and in contaminated soils, fluctuations in barometric pressure (caused by diurnal temperature variations and the passing of high- and low-pressure fronts) can cause rapid and substantial change in gas effluxes at the ground surface^34,35^. Through pressure gradients between the atmosphere and subsurface, periods of high barometric pressure inhibit upward migration of soil gas and release to the atmosphere, while periods of low barometric pressure enhance advective transport of soil gas to the ground surface^34–36^. “Barometric pumping” has been shown to be most relevant at sites with thick unsaturated zones^36^ (i.e. the partially water-saturated soils extending from the ground surface to the saturated groundwater zone), as is the case in multiple regions with active and potential oil and gas development in North America^37,38^ and Europe^39,40^. To our knowledge, the effect of barometric pumping is currently not considered when monitoring fugitive gas migration and CH_4_ emissions to the atmosphere.

Globally there are over 4 million onshore hydrocarbon wells^3^ and considering that well bore integrity issues were recently shown to be common^3,5^, it has become critical to effectively monitor the occurrence, magnitude and distribution of gas emissions at the ground surface. Without adequate monitoring, gas migration may not be detected and may pose an increased risk for aquifer contamination, explosive hazards and atmospheric greenhouse gas emissions^21,41^.

**Figure 1:**
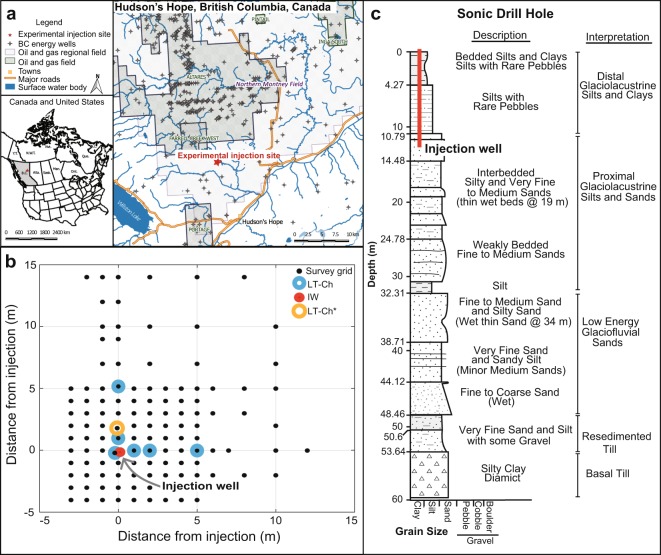
(**a**) Field site location in Northeastern British Columbia, Canada showing nearby oil and gas development and resource plays. (**b**) Monitoring network at the field site: black dots represent locations of survey chamber measurements, blue circles indicate locations of continuous measurements with long-term chambers (LT-Ch), and the red circle represents the injection well (IW). The long-term chamber from which the highest CH_4_ effluxes were measured (Fig. 2) is indicated by (LT-Ch*). (**c**) Core log collected from a sonic drill hole at the field site, outside of the monitoring grid. The depth of the natural gas injection well (12 m bgs) is indicated with a red line. The glacio-lacustrine deposits at the scale of the injection are comprised primarily of low-permeability silts.

The objective of our study was to monitor and evaluate the effect of barometric pressure changes on the temporal and spatial distribution of CH_4_ emissions to the atmosphere. Subsurface wellbore leakage was simulated through a controlled gas release experiment at a field site in the Montney resource play of Northeastern British Columbia, Canada. Since the 1950’s, there has been extensive oil and gas development in this portion of the Western Canadian Sedimentary Basin. At our field site, 30 m^3^ of natural gas was continuously injected 12 m below the ground surface (bgs) over a period of five days. The gas was injected into 60 m thick unsaturated, relatively homogeneous, distal glacio-lacustrine deposits. The depth and rate of well leakage are key factors that influence fugitive gas migration^2^. Here, we controlled these variables in order to focus on the effects of barometric pumping. To evaluate if and to what extent barometric pressure fluctuations affect the evolution of CH_4_ emissions at the ground surface, barometric pressure and CH_4_ effluxes were continuously measured at the field site for a total of 24 days during and after gas injection. Methane efflux was measured with an array of long-term and survey dynamic closed (non-steady-state) chambers connected to two gas analyzers (Fig. 1). This measurement strategy allowed evaluation of both the temporal evolution as well as the spatial extent of CH_4_ effluxes at the ground surface.

## Results

### Methane effluxes respond to barometric-pressure changes

**Figure 2:**
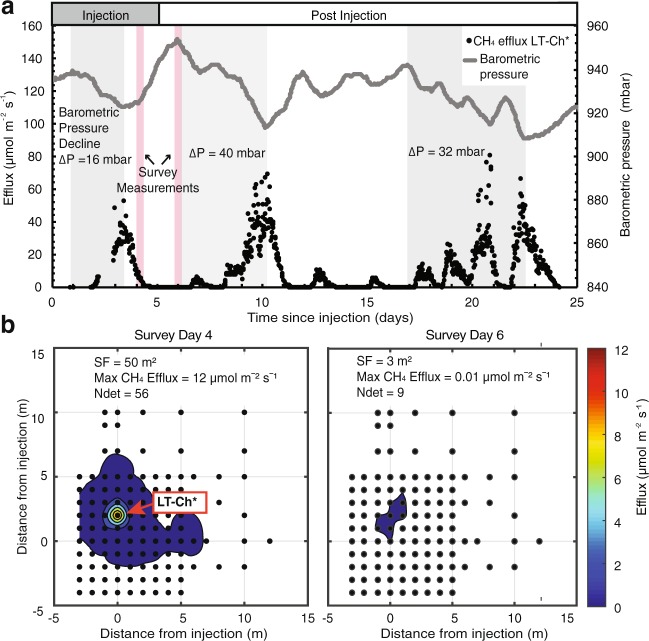
(**a**) Time series of barometric pressure and CH_4_ efflux, from the long-term chamber (LT-Ch*) with the highest fluxes during and post subsurface natural gas injection. Grey bars represent periods of decreasing pressure (>15 mbar in <5 days) over the duration of monitoring. Pink bars denote the two selected days when survey measurements were taken, to demonstrate the spatial response in effluxes from changes in barometric pressure. (**b**) Contour plots of surficial efflux footprints based on survey efflux measurements on Day 4 and 6; marking the end of periods with declining and increasing barometric pressure, respectively. SF = surficial efflux footprint, N_det_ = number of locations with detectable CH_4_ emissions.

Over the 24-day measurement period, barometric pressure varied between 909 to 951 mbar, while CH_4_ effluxes fluctuated from non-detect (<0.01 µmol m^−2^ s^−1^) to 80.8 µmol m^−2^ s^−1^ (112.0 g CH_4_ m^−2^ d^−1^). Figure 2. presents CH_4_ effluxes from the long-term chamber located where the highest fluxes (LT-Ch*) were measured. Increases and decreases in CH_4_ effluxes coincided directly with the fall and rise in barometric pressure, respectively (Fig. 2 and Supplementary Fig. S1). In particular, the three largest efflux events, with maximum values of 53.1, 69.5 and 80.8 µmol m^−2^ s^−1^ (73.6, 96.3, and 112.0 g CH_4_ m^−2^ d^−1^), all occurred when barometric pressure declined by more than 15 mbar in less than five days (Fig. 2). Although CH_4_ effluxes were lower at other long-term measurement locations, the release patterns and relation to barometric pressure fluctuations were comparable (Supplementary Fig. S2). The spatial extent of surface CH_4_ emissions also correlated with barometric pressure changes. During intervals of increasing barometric pressure, effluxes were at times non-detect in the entire study area, while during intervals of decreasing pressure, measurable effluxes occurred in areas greater than 50 m^2^ (e.g. Day 6 and Day 4) (Fig. 2).

An increase in total gas pressure and CH_4_ concentrations from a natural gas release in the unsaturated zone is expected to contribute to a continuous CH_4_ flux towards the ground surface comprised of advective and diffusive components. However, our experiment shows that barometric-pressure fluctuations can substantially modulate the CH_4_ emission patterns. Under intervals of increasing barometric pressure, air enters the soil profile from the atmosphere, leading to a downward displacement of soil gas^36^. This effect was observed through an inhibition or cessation of CH_4_ effluxes, even during the period of active injection (Day 1–5). During intervals of decreasing barometric pressure, soil gas is displaced upwards^36^. This was evident through the enhancement of CH_4_ effluxes^34^, continuing even after the injection stopped (Day 6–24). Although not reported for fugitive gas migration from oil and gas wells, the effect of barometric pressure on gas transport has been observed in the context of landfills^35,42–44^, soil vapor intrusion^45^, and peatlands^46^, consistent with our findings. These studies also report that a decline in barometric pressure triggers a rapid release of gas to the ground surface, at times causing a 35-fold variation in CH_4_ emissions day-to-day^35^ or, increases by up to 2 orders of magnitude over 10 min^46^.

### Evaluation of the effect of precipitation, atmospheric temperature and CH_4_ oxidation

Pulses in soil moisture content due to precipitation can displace gas and lead to rapid, short-lived fluxes to the ground surface^47,48^. On the other hand, because gas-phase molecular diffusion is 10,000 times faster than aqueous diffusion, an increase in soil moisture content by infiltration of water can reduce diffusive gas transport^49^. During our experiment, three precipitation events occurred, each lasting two days (Supplementary Fig. S2). Precipitation started when barometric pressure decreased and continued as barometric pressure increased. Total precipitation during these events was minimal (<2 mm per event), with the exception of a 16 mm rainfall event on Day 4. On Day 4, CH_4_ effluxes were decreasing prior to the precipitation event in response to an increase in barometric pressure starting on day 3 (Supplementary Figs. S1 and S2). Although an increase in soil moisture from precipitation may have contributed to lower effluxes through reduced diffusive gas transport, the temporal evolution of effluxes were not consistently related the occurrence of precipitation events. Soil and atmospheric temperature can enhance or decrease gas fluxes to the ground surface^51^. In our experiment, atmospheric temperature varied between −8.9 and 21.4 °C. The highest CH_4_ effluxes occurred when barometric pressure was decreasing and the temperature was 5.2, 2.9, and −2.2 °C (Supplementary Fig. S3), indicating no clear association between boundary layer air temperature and the magnitude of CH_4_ effluxes. Microbial oxidation of CH_4_ in the vadose zone can also contribute to a decrease in emissions at surface^16,50^. Typically, CH_4_ effluxes follow an exponential decline over time as the capacity for CH_4_ oxidation in the vadose zone progressively increases^51^. Oxidation may have contributed to lower CH_4_ effluxes in our experiment over time. However, changes in barometric pressure still had a more pronounced effect on surface emissions, leading to the highest CH_4_ effluxes towards the end of the experiment (Fig. 2). In summary, although precipitation events, variation in air temperature and an increase of CH_4_ oxidation capacity in the soil may have modulated CH_4_ effluxes, the effects were small and showed no clear relation with CH_4_ effluxes, which were regulated predominately by atmospheric pressure fluctuations.

### Effect of subsurface lithology

Gas migration in the unsaturated zone is influenced by subsurface lithology^52^. The unsaturated zone at our field site extends to a depth greater than 60 m below ground surface. A core log collected prior to installation of the injection well demonstrates that the deposits at the scale of the injection are formed by two distinct glacial outwash units: a 10 m thick layer of distal glacio-lacustrine silts and clays (comprised of approximately 5 m of bedded silts and clays transitioning into silt with rare pebbles) underlain by proximal glacio-lacustrine silts and sands (with gas injection occurring in a layer of interbedded silty and very fine to medium sands at 12 m depth, Fig. 1). While the deposits reveal continuous lithological conditions with vertical gradation from lower permeability units overlying higher permeability soils, there are subtle variations in grain sizes within units (Supplementary Fig. S4).

Fine grained, low permeability sediments are not unique to glacio-lacustrine settings, they are also common in marine, glacial, and fluvial sequences encompassing many regions of oil and gas development. Low-permeability near-surface material promotes lateral gas transport and limits vertical gas migration to the ground surface to discrete locations where “geologic windows” are present (e.g. fractures or regions of higher permeability)^52–54^. For example, at two separate landfill sites, soil gas with elevated CH_4_ was measured 35 m^55^ and 90 m^56^ from the landfill. In terms of barometric pumping, preferential pathways in low permeability sediments can have a greater effect on gas transport by allowing atmospheric air to infiltrate deeper into the subsurface^34,36^.

Although major geological structures could not be identified in the quaternary deposits at our field site, efflux data collected with survey chambers on a dense grid (Fig. 1) suggests that preferential pathways led to the formation of a discrete hot spot, where the highest CH_4_ effluxes were measured (LT-CH*, Fig. 2). Effluxes at all other measurement locations were lower than at the hot spot, however they also responded to changes in barometric pressure (R^2^ = 0.99, Supplementary Fig. S1). The variation in magnitude of effluxes between all seven long-term chambers demonstrates that the effect of barometric pumping on gas migration is influenced by site-specific conditions and permeability distribution (Supplementary Fig. S2). To this end, the spatial extent of CH_4_ emissions also varied as a result of forced lateral gas migration during high-pressure intervals and preferential gas migration to the surface during low-pressure intervals (Fig. 2). The effect of barometric pumping increases with the thickness of the unsaturated zone. A deeper vadose zone propagates greater pressure gradients between the atmosphere and subsurface^36^. The deep vadose zone in our study likely caused gas transport to be more sensitive to barometric pressure changes compared to other recent field studies on fugitive gas migration^15,16^. A water table >30 m below ground surface can occur in many areas of oil and gas development, and is not restricted to semi-arid or arid regions. For example: McKean and Venango counties within the Marcellus formation in Pennsylvania; Douglas and Elbert counties within the Wattenberg Gas Field, Colorado^37^; and the Peak District in the Lower Bowland unit, United Kingdom^39^ commonly have deep water tables.

### Quantitative analysis of barometric pressure-CH_4_ efflux correlation

**Figure 3:**
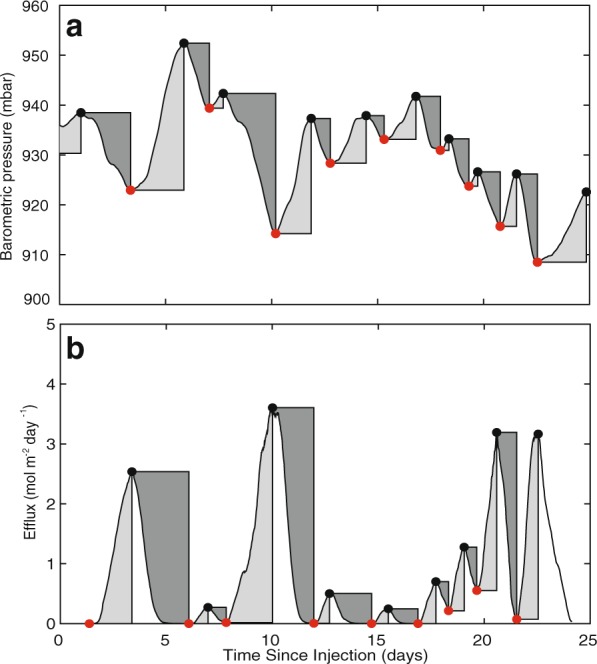
(**a**) Barometric pressure with time and (**b**) CH_4_ effluxes from LT-Ch* with time. Cumulative pressure and flux deviations during intervals with increasing barometric pressure are shaded in light grey, while cumulative pressure and flux deviations during intervals with decreasing barometric pressure are labeled in dark grey. Red dots mark lowest values, and black dots highest values for each interval for both barometric pressure and LT-Ch* CH_4_ efflux. For each interval the cumulative flux deviation (mol m^−2^) was correlated to the cumulative pressure deviation (mbar day) (see Fig. 4).

**Figure 4:**
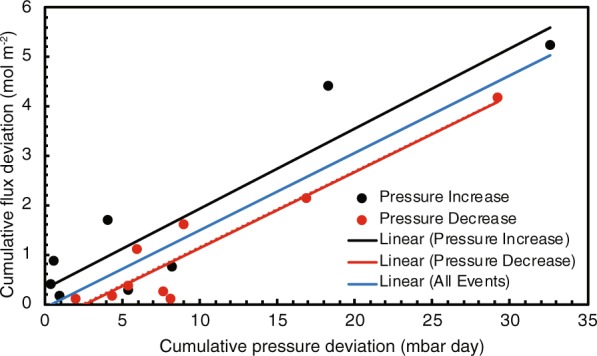
Cumulative pressure deviation and cumulative flux deviation for LT-Ch*. Cumulative pressure deviation-increases are shown in black dots and are correlated to cumulative flux deviation-decreases (R^2^ = 0.86). Cumulative pressure deviation-decreases are shown in red dots and are correlated to cumulative flux deviation-increases (R^2^ = 0.89). All events are also strongly correlated (R^2^ = 0.82). Correlations demonstrate that the cumulative flux deviation is dependent on the amplitude and duration of pressure changes.

Our results show that the combined effects of amplitude and length of barometric-pressure changes directly influence the occurrence and magnitude of CH_4_ effluxes. To quantitatively assess the cause and effect, we segregate the data record for LT-Ch* into intervals of continuous increasing or decreasing barometric pressure (Fig. 3). For intervals of decreasing barometric pressure, the “cumulative flux deviation” provides a quantitative measure for the time-integrated increase in CH_4_ efflux above the initial measured flux for the interval. Cumulative flux deviations are strongly correlated (R^2^ = 0.86) to cumulative pressure deviations, which represents a quantitative measure of the time-integrated decrease of barometric pressure below the initial measurement of the interval (Figs. 3 and 4). Similarly, cumulative flux deviations for decreasing CH_4_ effluxes were strongly correlated (R^2^ = 0.89) to cumulative pressure deviations for intervals of increasing barometric pressure (Figs. 3 and 4). These correlations support the notion that the magnitude and duration of a pressure cycle largely control the occurrence and extent of CH_4_ effluxes. The data provide strong evidence for two key controlling parameters where: (1) the magnitude of the pressure deviation (either increase or decrease) influences the pressure gradient that controls advective gas transport into or out of the subsurface and; (2) the duration of the pressure deviation affects the extent and volume of the surface-efflux event.

In addition to the magnitude and duration of a pressure cycle, our results indicate that the pattern of antecedent pressure variations affects effluxes. The cumulative pressure increases occurring in close sequence (Day 11–16) led to an accumulation of natural gas in the shallow subsurface. Stored gas was then released during multiple intervals of barometric pressure declines, leading to CH_4_ effluxes at the ground surface starting on Day 17 (after gas injection had stopped), followed by a period of more sustained and elevated CH_4_ emissions (Fig. 2).

## Discussion

Through a controlled natural gas release into a deep vadose zone, we demonstrate that the magnitude and duration of barometric pressure changes directly influence fugitive gas effluxes. Although our experiment was conducted over a relatively short time period (24 days), utilizing seven long-term chambers and a survey monitoring grid covering an area of ~180 m^2^, we were able to observe a direct correlation between barometric pressure changes and CH_4_ emissions from the vadose zone.

Despite monitoring with high spatial and temporal resolution, we may not have captured all dynamics of effluxes at the ground surface, due to the inherent complexity of fugitive gas migration in the subsurface^19–21^. Numerous factors including subsurface lithology, moisture content, and temperature influence the fate of fugitive gas and, compounding effects of these factors can lead to unexpected spatiotemporal variability in effluxes^16,19,33^.

For example, lithology primarily controls the distribution of gas (e.g. dense sedimentary layers cause lateral spreading of gas, while more permeable media facilitate vertical gas transport)^49^. While it is accepted that lithology is a key governing factor for the fate of fugitive gas, our results clearly show that changes in barometric pressure can substantially affect the magnitude and rate of effluxes. Changes in barometric pressure can enhance or inhibit effluxes by altering the pressure gradient driving subsurface gas transport between the atmosphere and subsurface^36^. In particular, we observed that at sites with a deep vadose zone, a decline in barometric pressure can trigger sudden, short-lived releases of gas even through relatively low permeability sediments. While lithology and subsurface structure led to select hot spots with high CH_4_ effluxes, barometric pressure changes had a dominant influence on the temporal distribution of CH_4_ emissions and also on its spatial extent.

To limit the uncertainty associated with monitoring fugitive gas, hydrogeological conditions and long-term barometric pressure trends should be considered when designing measurement methods and devising regulations. In addition, to accurately capture the occurrence and magnitude of CH_4_ emissions, monitoring should incorporate high resolution flux monitoring methods in space and time.

Globally there are few regulations to monitor fugitive-gas migration at oil and gas well pads. Those that do exist rely on sparse measurements taken around the wellbore^28–32^. For example, in the United States, the largest global producer of natural gas (~932 billion m^3^ produced in 2015)^57^, Pennsylvania is the only state that requires a soil-gas field survey to identify the concentration and areal extent of fugitive gas at a well pad with suspected gas migration. However, there are no requirements for how the survey should be conducted^31^. For Russia and Iran, the second and third largest producers of natural gas (~644 and 258 billion m^3^ produced in 2015^57^), there is little data available on the occurrence of gas migration or regulations to monitor and detect fugitive gas. In Canada, where this study took place and which is the world’s fifth largest producer (~191 billion m^3^ produced in 2015^57^), regulations to detect and monitor fugitive gas at well pads are enforced provincially. British Columbia, Alberta, Newfoundland and Saskatchewan all require gas migration testing under specific circumstances^28–30,32^. However, British Columbia is the only province that enforces monitoring. Monitoring will be conducted, if there are visual, auditory or olfactory indications of gas migration. In the case of confirmed gas migration, there are specific guidelines on how to conduct a shallow soil gas survey around the wellbore^32^.

Although current regulations aim to monitor fugitive-gas migration, they are focused on one-time survey measurements in close proximity to the well head, effectively only providing a “snapshot” of conditions at the site. Our results show that such intermittent and spatially restricted survey measurements could over- or under-estimate fugitive gas migration at well pads depending on the prevailing barometric pressure regime and the recent pattern of fluctuations preceding measurements. Further, our analysis on cumulative flux and pressure deviation show that subsurface storage and barometric pressure fluctuations can lead to conditions that allow CH_4_ effluxes to occur after a leaking well has been repaired, depending on the depth of the leak and mass of gas released. These results could be of particular importance for monitoring abandoned and orphaned wells.

## Conclusions

The frequency, amplitude and duration of barometric-pressure cycles directly controlled the magnitude and spatiotemporal variability of CH_4_ effluxes from a natural-gas release into a deep unsaturated zone. During periods of increasing barometric pressure, CH_4_ effluxes declined, at times below the detection limit. However, when barometric pressure decreased, CH_4_ effluxes rapidly increased, at times greater than 20-fold in less than 24 hours. The results indicate that barometric-pressure fluctuations can affect gas transport to the ground surface at sites with a deep-water table. The dynamic response in effluxes to changes in barometric pressure demonstrates the sensitivity and difficulty in effectively detecting fugitive gas migration and accurately estimating emissions to atmosphere. Currently, the potential effects of barometric-pumping are not considered when assessing the occurrence or magnitude of gas migration around oil and gas sites. Our results provide a framework to better understand, target and constrain fugitive gas migration at oil and gas well pads and will help mitigate risks for aquifer contamination, explosive hazards and atmospheric greenhouse gas emissions^21,41^.

## Methods

### Natural gas injection

The experiment took place in the fall from September 26^th^ to October 20^th^, 2017. For five days a total of 30 m^3^ of natural gas (93.8% CH_4_, 1.8% C_2_H_6_, 0.2% C_3_H_8_, ~0.01% C_4+_, 3.0% N_2_, 0.3% CO_2_, 0.9% O_2_ at standard conditions for temperature and pressure (STP), 273.15 K (0 °C, 32 °F) and absolute pressure of 10^5^ Pa (100 kPa, 1 bar)) was injected at 12 m depth in unsaturated glacio-lacustrine deposits with a water table greater than 60 m below ground surface. Gas was injected from canisters connected (via ¼” ID polyethylene tubing) to an in-line electronic mass flow controller (Red-y smart GSC-C9SA-BB26) and a vertical injection well (½” ID polyethylene tubing). The injection rate (7 m^3^ d^−1^) was controlled with mass flow controller software (Get Red-y, Vögtlin Instruments AG, Switzerland). The rate was selected based on average reported surface casing vent flows from Alberta and British Columbia, Canada^2^, assuming that leaky wells could lead to gas migration of similar magnitude.

### Soil-gas efflux measurements

Water vapor and CO_2_ effluxes were monitored continuously with seven long-term dynamic closed chambers (LI-8100-104, LI-COR Inc., Lincoln, NE) operated with a CO_2_ infra-red gas analyzer (IRGA) (LI-8100, LI-COR Inc., Lincoln, NE). To simultaneously measure CH_4_ with CO_2_ effluxes, the IRGA was coupled with an extended range (0.01 to 100,000 ppm) Ultraportable Greenhouse Gas Analyzer (UGGA, Los Gatos Research Inc., Mountain View, CA). Each chamber was connected to a multiplexer (LI-COR LI-8150, LI-COR Inc., Lincoln, NE) to allow all seven chambers to autonomously alternate and periodically collect data approximately every 25 min. Our reported minimum detectable flux (MDF) for CH_4_ is 0.01 µmol m^−2^ s^−1^ given a Δc = 0.2 ppm. The manufacturer UGGA analytical accuracy is <2ppb (1 sec), 100 times smaller than the minimum Δc we use to calculate an efflux, providing confidence in our MDF. The spatial distribution of effluxes was measured over a monitoring grid including up to 123 locations for 13 sampling events (Fig. 1). For the survey, a dynamic closed (non-steady-state) chamber (LI-8100-103, LI-COR Inc., Lincoln, NE) was connected to an IRGA for CO_2_ and water vapor measurements and an extended range Greenhouse Gas Analyzer (Los Gatos Research Inc., Mountain View, CA) for CH_4_ measurements. All analyzers were powered by a 2000-Watt generator and a solar panel system installed by Empower Energy Corp (Kelowna, BC, Canada).

Both long-term and survey chambers were placed on pre-installed polyvinyl chloride (PVC) collars (20 cm ID) inserted 4 cm into the soil and covering an area of 317.8 cm^2^. Both long-term and survey chamber measurements were conducted for a period of 2 min 30 sec. Gas concentration increases within the chambers were monitored with the IRGA and UGGA instruments over this measurement period. Soil-gas effluxes (*F* in µmol m^−2^s^−1^ or g m^−2^ d^−1^) were calculated from the exponential increase in concentrations in the chamber over durations ranging from 45 sec to 80 sec. Effluxes are shown from one of the seven chambers where the largest effluxes and the most pronounced change from barometric pressure changes occurred (Fig. 2). However, all seven long-term chambers showed a similar response in effluxes to the fluctuations in barometric pressure (Supplementary Fig. S2). Background CH_4_ effluxes were monitored for five days before the injection and remained non- detectable during this time.

### Environmental monitoring

Barometric pressure and temperature were continuously recorded with a pressure transducer installed at the field site (vanEssen Instruments Baro-Diver, Kitchener, ON, Canada). Precipitation records were retrieved from the nearest weather station (Fort St. John Airport, BC, Canada).

### Data analysis

Barometric pressure and CH_4_ effluxes (for LT-CH*) were segregated into intervals of continuous increase or decrease (Fig. 3). We correlated time (t) integrals of pressure increase (dp) to time integrals of efflux decrease (dq) and, time integrals of pressure decrease (dp) to time integrals of efflux increase (dq) (Fig. 4). We calculated the integrated value of change in flux (dq), instead of the integrated value of flux (q) in order to account for flux changes that were specifically attributed to the corresponding barometric pressure change, and to minimize the effect of prior events on the analysis. Using this analysis, the time integral for the change in flux over the interval from *t*_*i*_ to *t*_*i*+1_ yields the cumulative flux deviation, which is proportional to the time integral of pressure difference, i.e. the cumulative pressure deviation:

$${\int }_{{t}_{{i}}}^{{{t}}_{{i}+1}}dq\,dt\,\alpha {\int }_{{t}_{{i}}}^{{{t}}_{{i}+1}}dq\,dt$$

This approach allows correlation of cumulative flux deviations to cumulative pressure deviations. In principle the approach assumes: soil gas pressure is at equilibrium with atmospheric pressure when the rate of pressure change is zero; the decline in soil gas pressure is negligible for each interval; effects from diffusion are negligible compared to advective fluxes; and that each efflux event is isolated without contributions from prior efflux events. Although these assumptions are unlikely fully satisfied, the fact that a strong statistical correlation between cumulative pressure deviations and cumulative flux deviations is found, re-emphasizes the dominating and immediate effect of barometric pressure fluctuations on CH_4_ emissions.

## Supplementary information

Supplementart Information
