# Supplementary material for: Barometric-pumping controls fugitive gas emissions from a vadose zone natural gas release
Source: Sci Rep. 2019 Oct 1;9:14080. doi: 10.1038/s41598-019-50426-3 (PMC6773692; doi:10.1038/s41598-019-50426-3)
Supplement: Supplementary file 1 — Supplementart Information [file 41598_2019_50426_MOESM1_ESM.pdf]

# Supplementary Information for

## Barometric-pumping controls fugitive gas emissions from a vadose zone natural gas release

Olenka N. Forde, Aaron G. Cahill, Roger D. Beckie and K. Ulrich Mayer

Corresponding author: Olenka N. Forde
Email: oforde@eoas.ubc.ca

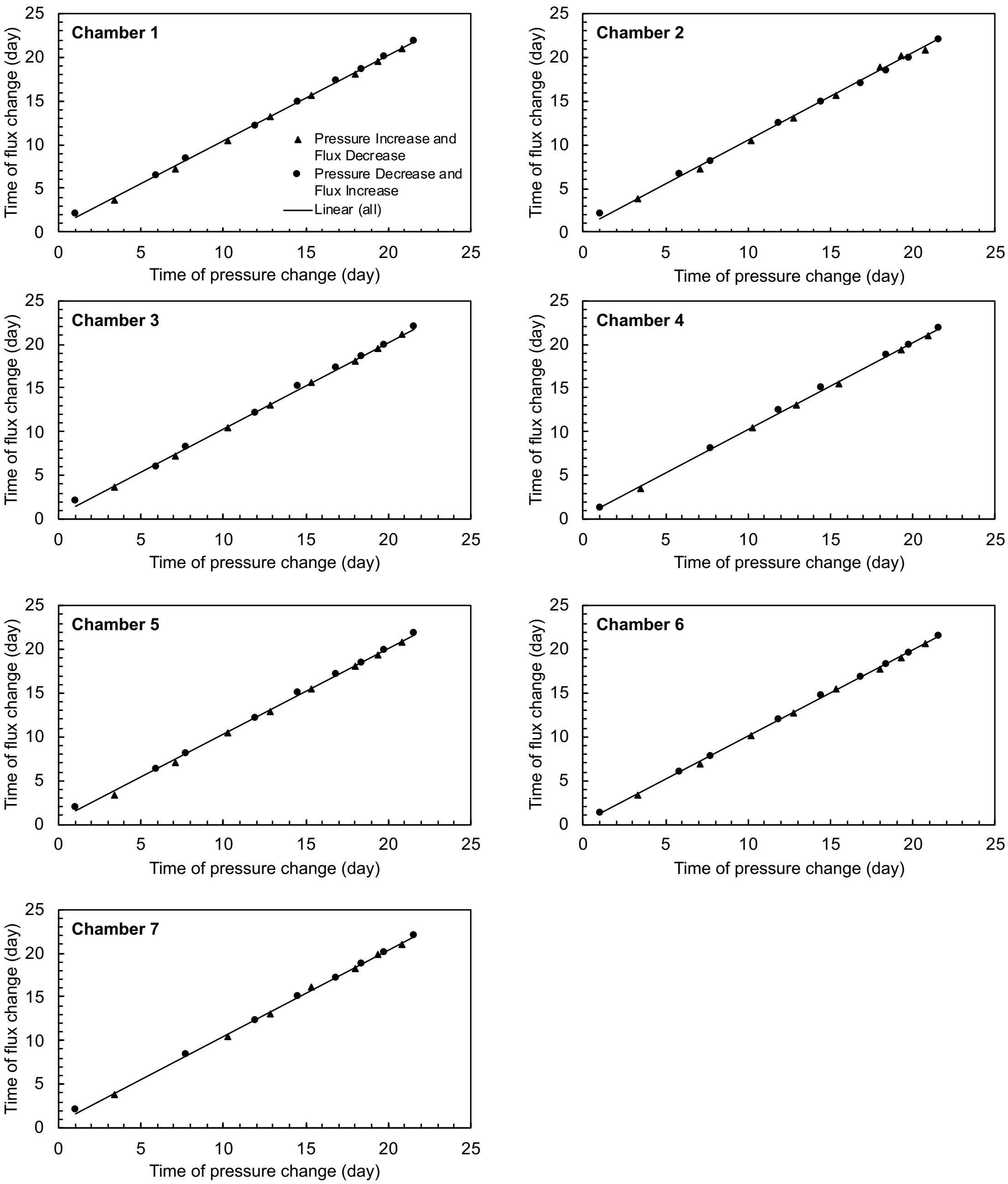


**Figure S1.** Time of pressure change plotted against time of flux change for long-term chambers 1 to 7. For all chambers there was a strong correlation ($R^2 = 0.99$) between the time of the change in barometric pressure to the time of the flux increase or decrease. These correlations demonstrate that barometric pressure directly influences the occurrence of $CH_4$ effluxes at ground surface.

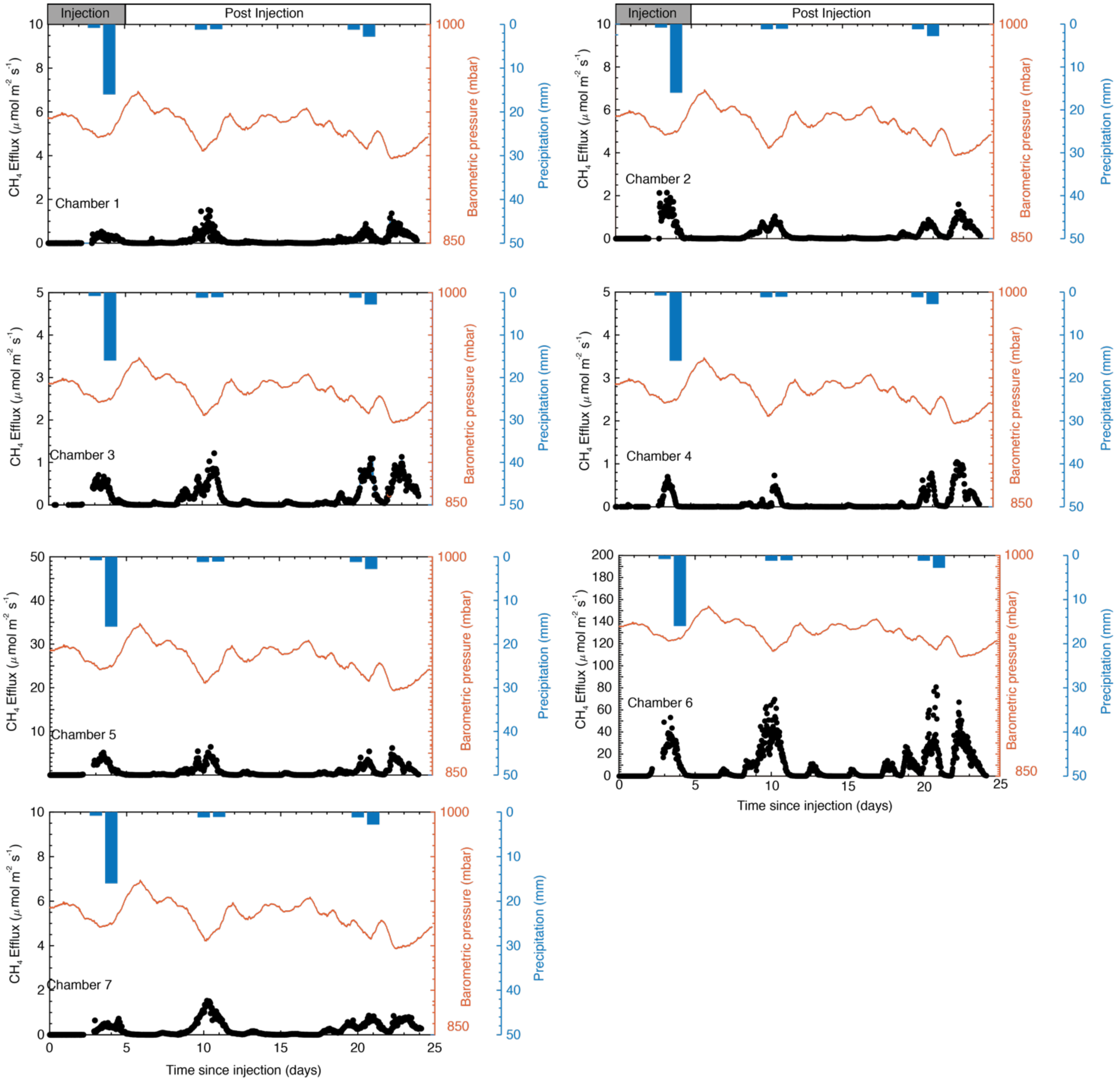


**Figure S2.** Precipitation, barometric pressure, and $CH_4$ effluxes (from long-term chambers 1 to 7) monitored over the duration of the experiment. Independent of the location, $CH_4$ effluxes all show the same temporal variation and response to increasing and decreasing barometric pressure (see also Fig. S1). Methane effluxes were already decreasing on Day 4, before the largest precipitation event, and increased during precipitation events between Days 21 and 22. These trends suggest precipitation did not consistently influence the magnitude of $CH_4$ effluxes.

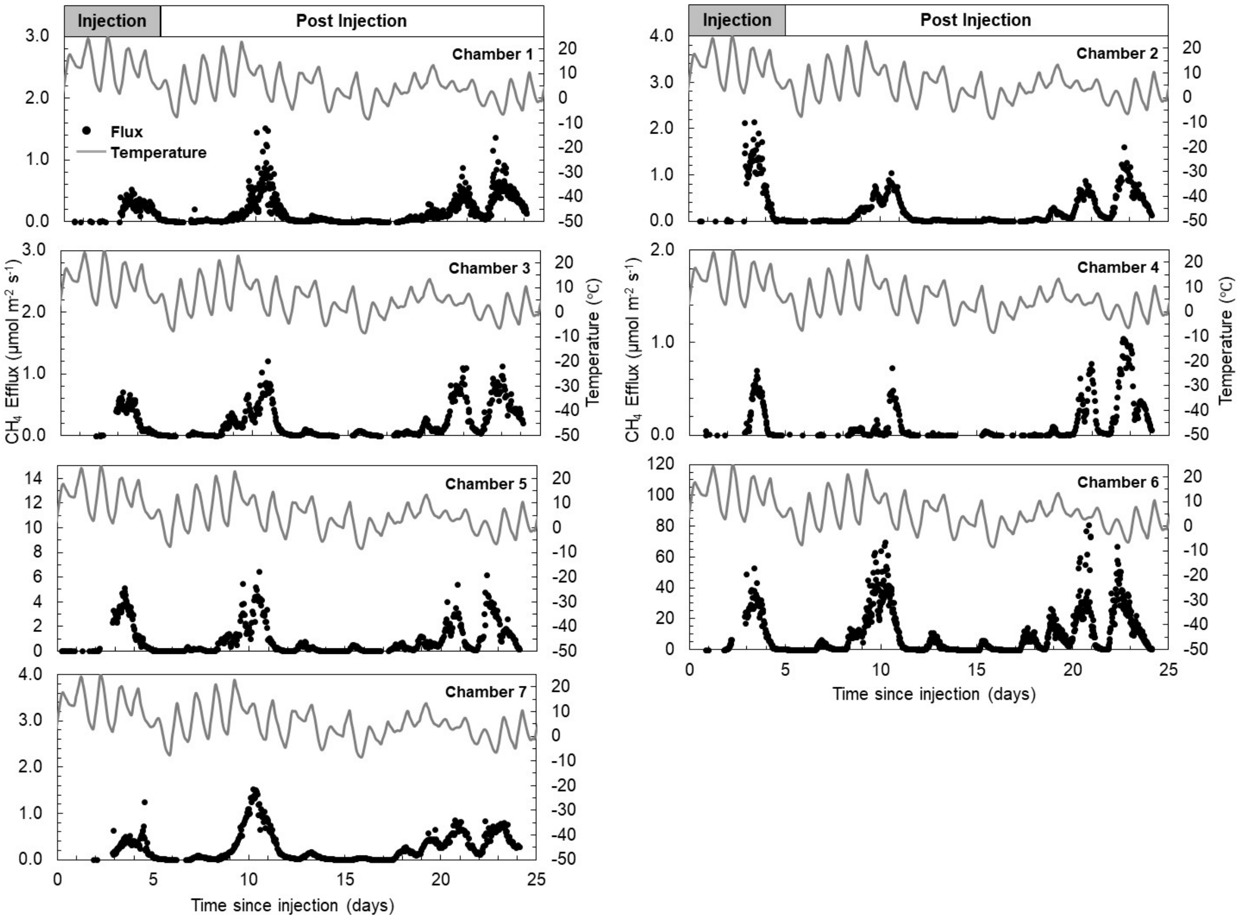


**Figure S3.** Methane effluxes from long-term chambers 1 to 7 plotted against atmospheric temperature measured at the field site. Although temperature declined over the duration of the experiment and showed pronounced diurnal fluctuations, $CH_4$ effluxes continued to fluctuate independently, with the highest effluxes at Chamber 6 on Day 22. These data show that temperature fluctuations did not influence the magnitude of $CH_4$ effluxes.

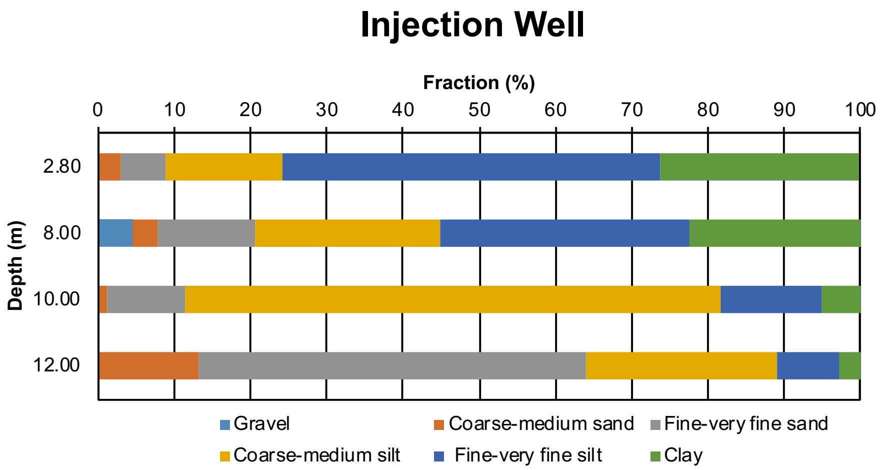


**Figure S4.** Grain size analysis from the well where gas was injected. Results show that the subsurface lithology is relatively homogeneous, dominated by silts and clay, with more fine sand 12 m bgs.
